# Supplemental Stimulation Improves Swing Phase Kinematics During Exoskeleton Assisted Gait of SCI Subjects With Severe Muscle Spasticity

**DOI:** 10.3389/fnins.2018.00374

**Published:** 2018-06-01

**Authors:** Andrew Ekelem, Michael Goldfarb

**Affiliations:** Department of Mechanical Engineering, Center for Rehabilitation Engineering and Assistive Technology, Vanderbilt University, Nashville, TN, United States

**Keywords:** spinal cord injury, spasticity, exoskeleton, functional electrical stimulation, locomotion, rehabilitation

## Abstract

Spasticity is a common comorbidity associated with spinal cord injury (SCI). Robotic exoskeletons have recently emerged to facilitate legged mobility in people with motor complete SCI. Involuntary muscle activity attributed to spasticity, however, can prevent such individuals from using an exoskeleton. Specifically, although most exoskeleton technologies can accommodate low to moderate spasticity, the presence of moderate to severe spasticity can significantly impair gait kinematics when using an exoskeleton. In an effort to potentially enable individuals with moderate to severe spasticity to use exoskeletons more effectively, this study investigates the use of common peroneal stimulation in conjunction with exoskeleton gait assistance. The electrical stimulation is timed with the exoskeleton swing phase, and is intended to acutely suppress extensor spasticity through recruitment of the flexion withdrawal reflex (i.e., while the stimulation is activated) to enable improved exoskeletal walking. In order to examine the potential efficacy of this approach, two SCI subjects with severe extensor spasticity (i.e., modified Ashworth ratings of three to four) walked in an exoskeleton with and without supplemental stimulation while knee and hip motion was measured during swing phase. Stimulation was alternated on and off every ten steps to eliminate transient therapeutic effects, enabling the acute effects of stimulation to be isolated. These experiments indicated that common peroneal stimulation on average increased peak hip flexion during the swing phase of walking by 21.1° (236%) and peak knee flexion by 14.4° (56%). Additionally, use of the stimulation decreased the swing phase RMS motor current by 228 mA (15%) at the hip motors and 734 mA (38%) at the knee motors, indicating improved kinematics were achieved with reduced effort from the exoskeleton. Walking with the exoskeleton did not have a significant effect on modified Ashworth scores, indicating the common peroneal stimulation has only acute effects on suppressing extensor tone and aiding flexion. This preliminary data indicates that such supplemental stimulation may be used to improve the quality of movement provided by exoskeletons for persons with severe extensor spasticity in the lower limb.

## Introduction

Spasticity is a common comorbidity resulting from spinal cord injury (SCI), where ~68–75% of individuals with SCI experience spasticity and 20–40% have problematic spasticity that restricts activities of daily living (Adams and Hicks, 2005). Spasticity is commonly defined as involuntary muscle activity initiated by uninhibited reflex circuits, and can be categorized as intrinsic tonic, intrinsic phasic, or extrinsic nocuous (Decq, 2003; Adams and Hicks, 2005). Tonic and phasic spasticity are thought to be caused by the hyperactive stretch reflex circuits after central neurologic impairment of the corticospinal inhibitory pathways. Symptoms of spasticity include a counter torque proportional to the angular velocity about the joint and an increase in muscle tone. Spasticity is ordinarily evaluated through the Modified Ashworth Scale (MAS) which involves a trained assessor rapidly advancing a joint through its range of motion and evaluating the stiffness of the joint on a scale from zero to four, see Table 1 (Bohannon and Smith, 1987; Biering-Sørensen et al., 2006). Significant tonic and/or phasic spasticity can be problematic for attempts to restore mobility to a paretic or paralyzed limb.

**Table 1:** Modified ashworth scale description (Bohannon and Smith, 1987).

**Modified ashworth scale**	**Description**
0	No increase in muscle tone
1	Slight increase in muscle tone, manifested by a catch and release or by minimal resistance at the end range of motion when the affected parties moved in flexion or extension
1+	Slight increase in muscle tone, manifested by a catch, followed by minimal resistance throughout the remainder (less than half) of the range of motion
2	More marked increase in muscle tone through most of the range of motion, but the affect part is easily moved
3	Considerable increase in muscle tone, passive movement is difficult
4	Affected part is rigid in flexion or extension

Spasticity may be managed to achieve a balance between useful and detrimental effects by progressing from physical rehabilitation, pharmacological intervention, injection, intrathecal baclofen, and surgery (Adams and Hicks, 2005). Useful effects of spasticity induced muscle activity include: reduced muscle atrophy and improved blood circulation, which can reduce predispositions to other comorbidities that commonly result from frequent wheelchair use (Demirel et al., 1998; Adams and Hicks, 2005). Therefore, spasticity is typically managed to a level that best balances comfort and quality of life (QOL).

Recently, lower limb exoskeletons have begun to emerge onto the commercial marketplace. Such devices can facilitate legged mobility for individuals with SCI. These devices have the potential to improve the QOL of people with lower limb paralysis by enabling them to walk, generally via actuated knee and hip joints (Contreras-Vidal et al., 2016). Exoskeletons have been shown to enable safe over-ground weighted walking and gait training for the mobility impaired, particularly for individuals with SCI (Contreras-Vidal et al., 2016). The presence of severe spasticity, however, can severely impair the ability of an exoskeleton to provide a viable walking movement (Esquenazi et al., 2012; Aach et al., 2013; Kolakowsky-Hayner, 2013). Severe extensor spasticity, in particular, can preclude hip and knee flexion during the swing phase of walking, thereby largely nullifying the ability of the exoskeleton to provide effective legged mobility.

Functional electrical stimulation (FES) applies artificial electrical impulses to initiate action potentials in muscle and nerves for a functional response. FES has proven effective in enabling ambulation in the SCI population, and promoting neural recovery for incomplete the SCI population (Graupe and Bazo, 2015; Young, 2015). Depending primarily on FES alone for gait can be metabolically taxing, is prone to rapid fatigue, and may lack limb control and stability. For these reasons effort has gone into combining FES with orthotic devices, generally called hybrid FES systems, which can provide several advantages relative to using FES alone (Andrews et al., 1988; Isakov et al., 1992; Goldfarb et al., 2003; Bulea et al., 2013; del-Ama et al., 2014; Chang et al., 2017; Anaya et al., 2018), and one prior study investigated a hybrid FES system that couples FES with a robotic exoskeleton (Ha et al., 2016). That study specifically coupled a lower limb exoskeleton with FES of the quadriceps and hamstrings muscle groups of each leg, and demonstrated reduced exoskeleton motor torque and power when used in a hybrid FES manner.

The intent of this research is to investigate the potential of supplementing a lower limb exoskeleton with FES for the purpose of enabling individuals with severe extensor spasticity to effectively walk in a lower limb exoskeleton when those individuals would otherwise have difficulty to do so. Preliminary studies indicate that exoskeleton walking may have beneficial effects with respect to mitigating spasticity (Esquenazi et al., 2012; Aach et al., 2013; Contreras-Vidal et al., 2016), and as such, making this technology available for individuals with moderate to severe spasticity may have particular value for this sub-population. In order to do so, the authors propose here to supplement an exoskeleton with stimulation of the common peroneal nerve with the intent of exciting the flexion withdrawal reflex. Common peroneal nerve stimulation has been shown to assist neurologically impaired individuals during the swing phase of gait by initiating a concerted flexion of the hip, knee, and ankle (Zehr et al., 1997; Bajd et al., 1999; Embrey et al., 2010; O'Dell et al., 2014; Street and Singleton, 2017). Stimulation of the common peroneal nerve activates the flexor withdrawal reflex, which comprises afferent neurons activating motor units responsible for flexion, as well as inhibitory interneurons that inhibit ipsilateral extensors while activating contralateral extensors (Sherrington, 1910). As such, the hypothesis behind peroneal stimulation is twofold: first, the reflex is hypothesized to temporarily inhibit the extensor tone resulting from severe extensor spasticity, and second, the reflex is expected to recruit the lower limb flexors, and therefore supplement the exoskeleton motors during swing. In order to evaluate this hypothesis, a lower limb exoskeleton system was configured to incorporate supplemental FES of the common peroneal nerve, and experiments were conducted on two SCI subjects with severe extensor spasticity to evaluate the effect of supplemental peroneal stimulation on exoskeleton-generated swing phase movement.

## Methods

### Clinical status

Two subjects were recruited for these experiments. The subjects met the inclusions criteria of having thoracic-level motor-complete SCI (i.e., ASIA A or B) to exclude voluntary motor control of the lower limbs; had right and left leg extensor spasticity rated on the MAS of three or greater in one or more joints; and were responsive to stimulation of the flexor withdrawal reflex. Subject characteristics relevant to the experiments are given in Table 2, and respective MAS scores given in Table 3. This study was conducted with the informed consent of each subject and with the approval of the Vanderbilt University Internal Review Board.

**Table 2:** Subject characteristics.

**Subject ID**	**S1**	**S2**
ASIA	T11 B	T4 B
Level	T11	T4-5
DOI	Mar 2010	Dec 2013
Body Mass (kg)	66	88
Height (m)	1.83	1.73

**Table 3:** Modified ashworth scale ratings for each subject.

**Muscle Group**	**S1 R/L**	**S2 R/L**
Hip flexors	1+/1	0/1
Hip extensors	2/1	0/0
Knee flexors	2/2	2/2
Knee extensors	3/3	3/3
Hip adductors	3/3	3/3
Hip abductors	0/0	0/0
Ankle dorsiflexors	0/0	0/0
Ankle plantarflexors	3/3	4/3

### Indego exoskeleton

**Figure 1:**
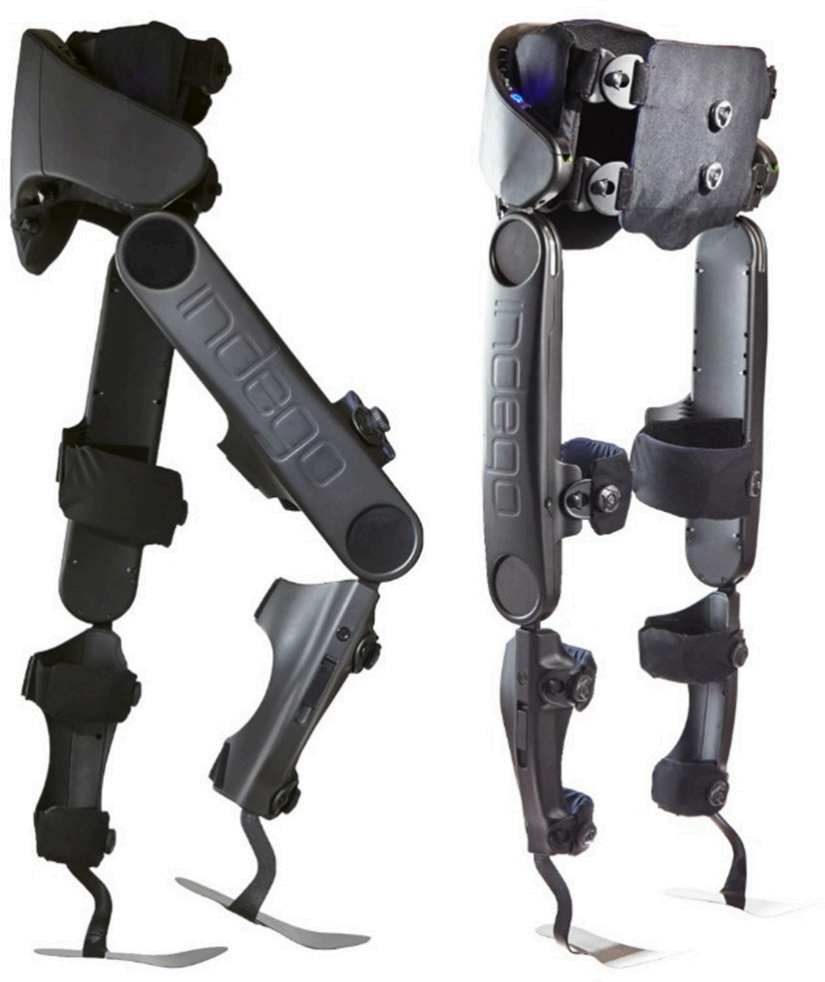
Indego exoskeleton. Photograph courtesy of Parker-Hannifin.

The walking controller was implemented on a lower-limb exoskeleton prototype, shown in Figure 1. The prototype utilizes a commercially-available lower-limb exoskeleton (Indego Exoskeleton, Parker Hannifin Corp) as a hardware platform, but replaced the commercial version of the software with experimental control software written by the authors—in this case, the walking controller with supplemental stimulation of the common peroneal nerve on each leg. The experimental control software was implemented in MATLAB Simulink/Stateflow (Mathworks Inc) through a controller area network (CAN) serial cable which was plugged into a CAN port in the exoskeleton. The Indego exoskeleton hardware platform incorporates four motors for powered movement of bilateral hip and knee joints in the sagittal plane, in addition to built-in ankle-foot-orthoses (AFOs) at both ankle joints to provide ankle stability and transfer the weight of the exoskeleton to the ground. Onboard electronic sensors include encoders at each joint that provide the respective joint angles and angular velocities, and a six-axis inertial measurement unit (IMU) in each thigh link, which provide the left and right thigh angles with respect to the vertical. The total mass of the exoskeleton including the battery is ~12 kg (26 lbs). Further details regarding the Indego exoskeleton functions can be found in (Quintero et al., 2011; Hartigan et al., 2015).

**Figure 2:**
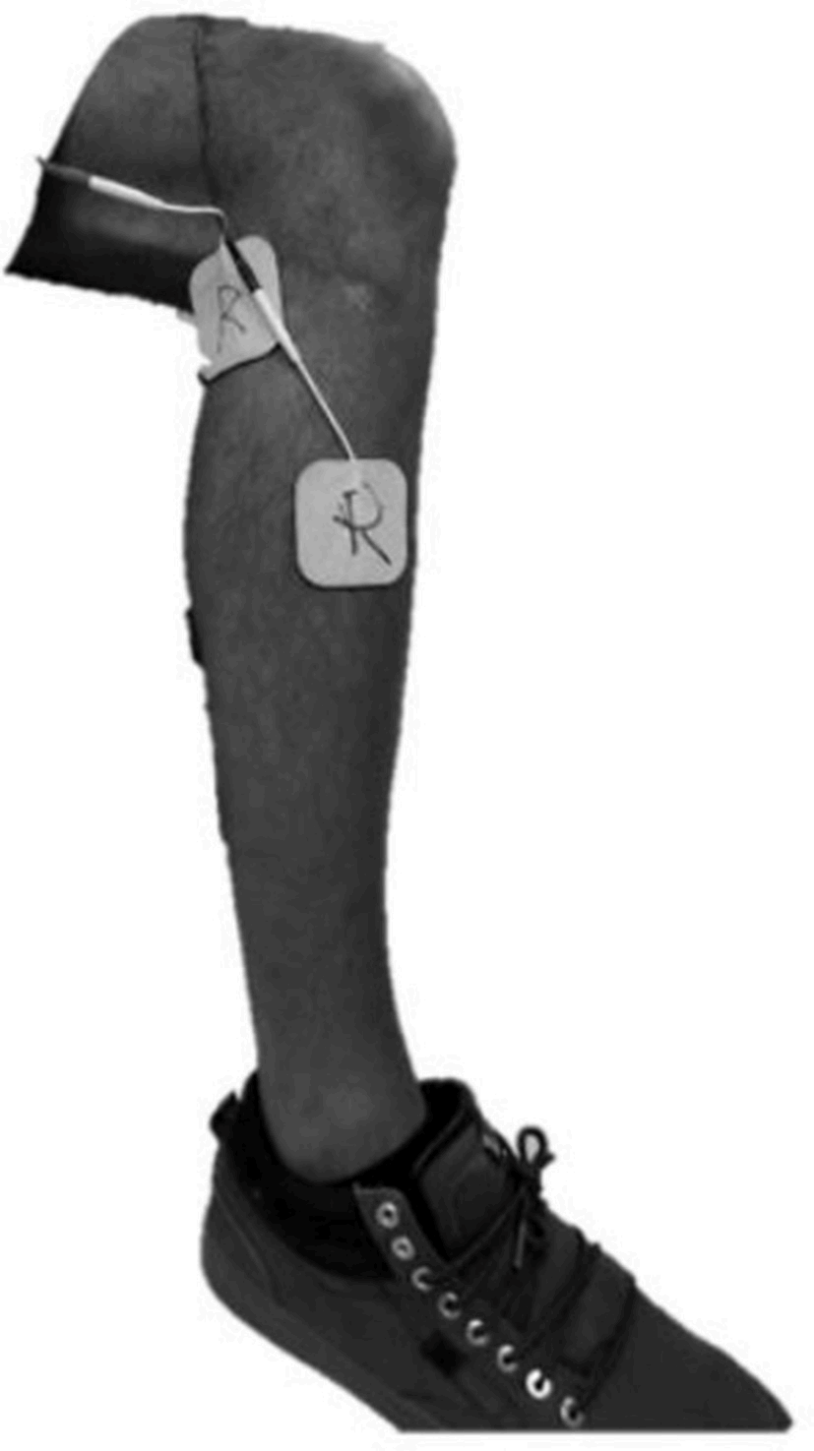
Typical electrode placement for stimulation of the common peroneal nerve.

A custom stimulator was developed on an embedded system, which was controlled in the same MATLAB Simulink/Stateflow environment used to control the exoskeleton, and connected via the same CAN cable. As such, the sensor information and control associated with the exoskeleton and stimulator were fully synchronized. The custom stimulator employed a charge-balanced bipolar stimulation waveform with 200 μs pulse widths at a frequency of 50 hz. The device controls the peak current output with a maximum amplitude of 80 mA. Square sticky-gel 3.8 x 3.8 cm (1.5 x 1.5 in) electrodes were placed over the common peroneal nerve on each leg, see Figure 2. One electrode was positioned over the fibula head, near the underlying common peroneal nerve, and the second electrode was over the tibialis anterior, ~4 cm below the tibial tuberosity. Surface electrodes produce a diffuse electric fields so precision (<1 cm) is not necessary. This electrode arrangement avoided significant eversion of the ankle while enabling the flexor withdrawal to be sufficiently activated. A total of two stimulator channels were used, one for each leg. The stimulation amplitude was set prior to donning the exoskeleton to a level that elicited strong ankle dorsiflexion, but not so high as to raise the heel off the ground while in a seated position. The stimulation amplitude for the subjects, respectively, were set to 28 and 44 mA on the right, and 28 and 54 mA on the left.

### Hybrid controller

**Figure 3:**
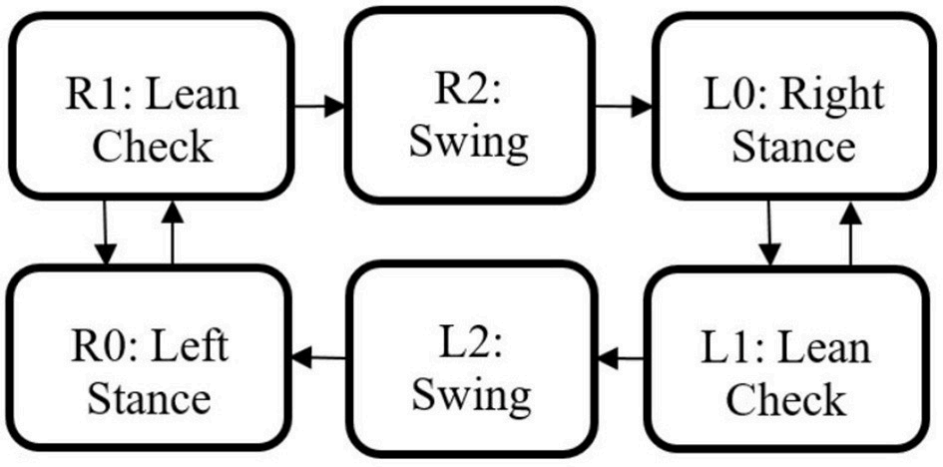
Walking state machine with corresponding transition conditions in Table 4.

**Figure 4:**
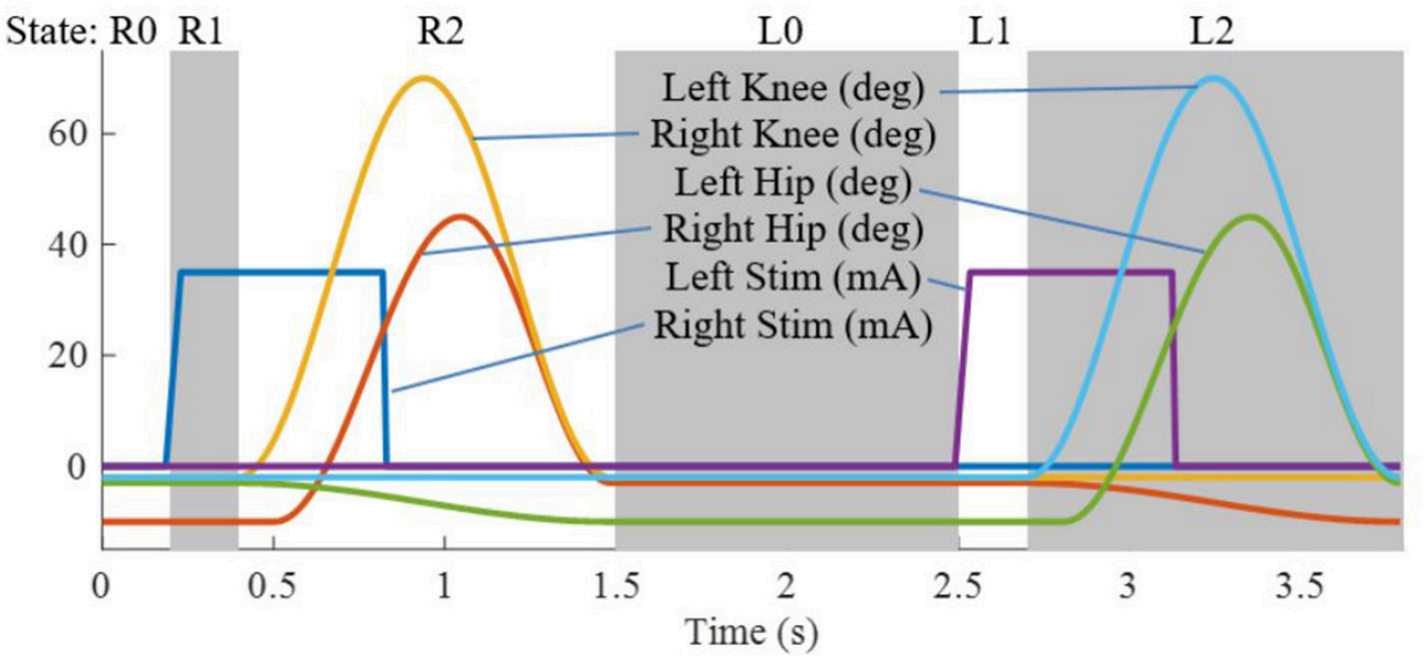
Trajectories for the four exoskeleton joints and the stimulation amplitude for the right and left legs as the state machine progresses through two steps.

The state machine for the Indego walking controller is shown in Figure 3 with state transition conditions described in Table 4. To account for the latency of the stimulation to produce movement, the stimulation was activated during the lean check state, which identifies the user's intent to take a step by maintaining a forward lean (i.e., forward movement of the estimated center of pressure), as measured by inertial sensors in the exoskeleton, for 200 ms. Once swing is initiated, the stimulation remains on for 40% of the swing phase, during which the majority of flexion occurs. Figure 4 shows the desired joint trajectories commanded by the exoskeleton controller as the state machine cycles through a right and left step, along with the corresponding timing of the stimulation pulse. When the stimulation is off, the stimulation amplitude is maintained at zero.

**Table 4:** State machine transition conditions.

**Transition**	**Condition**
R0 to R1 or L0 to L1	Rear thigh angle exceeds lean threshold
R1 to R0 or L1 to L0	Rear thigh angle less than lean threshold
R1 to R2 or L1 to L2	State timer exceeds 200 ms
R2 to L0 or L2 to R0	State timer exceeds 1.1 s

### Experimental procedure

**Figure 5:**
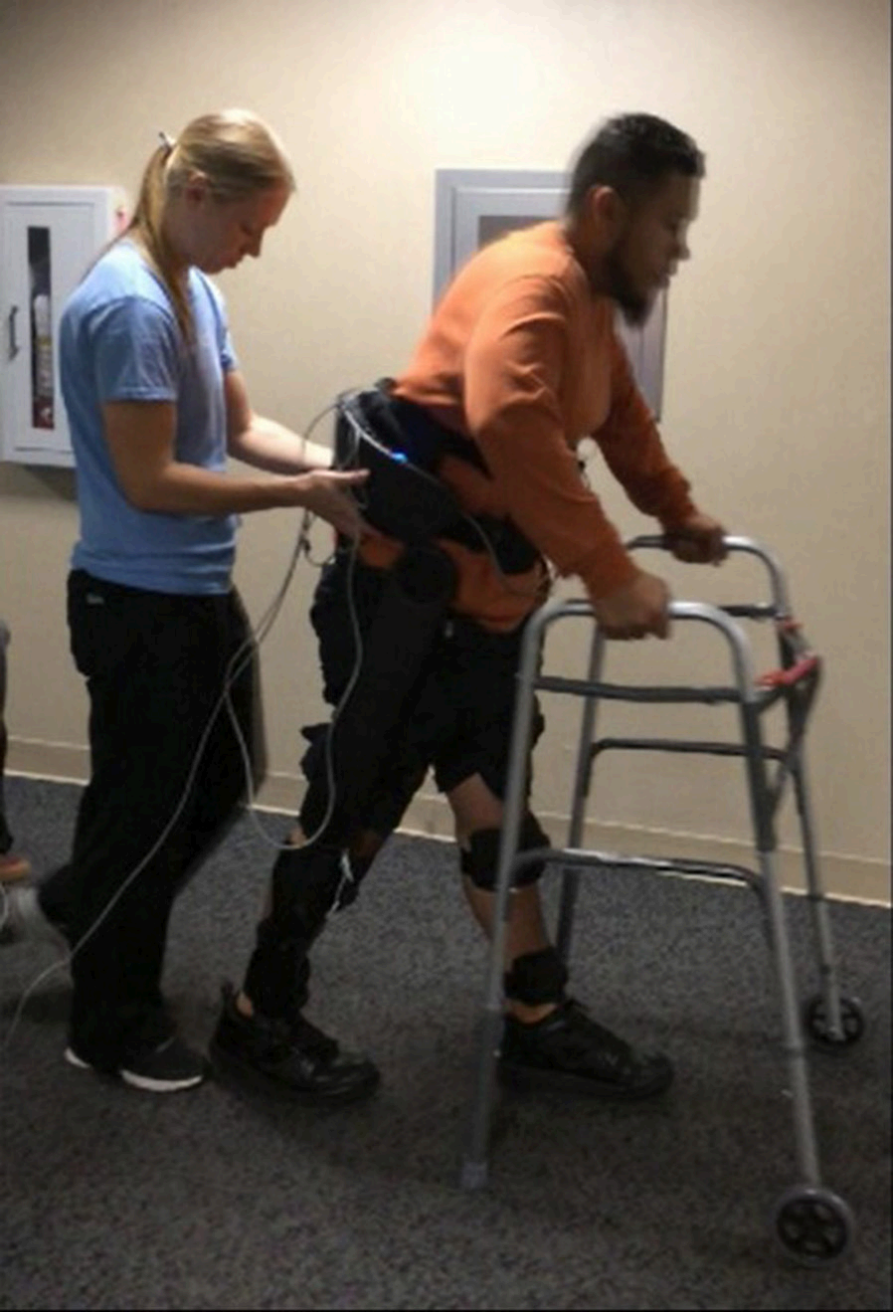
Subject walking with the exoskeleton and stimulator, using a walker for balance. The physical therapist monitors the gait as a precaution in accordance with the Vanderbilt IRB. The subject has consented to the use of this photograph.

Participants were fit with the exoskeleton and given practice sessions (<4 h per day) walking with FES on and off, until they could comfortably walk, and advance the balance aid (i.e., the walker), see Figure 5, for 1 h taking breaks as needed. The practice sessions were scheduled approximately a week apart and did not have an appreciable effect on the subjects' spasticity during any term of the study. After sufficient training sessions, each subject had a data session where they donned the FES system, Indego, and XSens MVN Awinda system (Xsens Technologies B.V., Netherlands) motion capture system and walked for ~1 h, taking rest breaks as needed. The Xsens wireless sensor based motion tracking system was used to capture the kinematic data of the lower limbs during each session. This system includes seven sensors, one sensor for each limb segment (two feet, two shanks, two thighs, and one hip). The sensors were orientated as recommended by Xsens except the thigh sensors were placed medial rather than lateral as to not interfere with the exoskeleton. The Xsens system was calibrated in stance before each 6 min walk session. Data was saved every 6 min as to not overload the computer and risk a loss of data. Neither the environment nor exoskeleton created substantial interference with the Xsens measurement, as validated via Xsens calibration procedures. The trajectories measured by Xsens motion tracker were then processed in MATLAB to identify the peak flexion angle of each joint for each step. For these experiments, the stimulation was programmed to alternate on and off every 10 steps to assess the relative value of stimulation on exoskeleton motion. Gait data was recorded during the data session, totaling 232 steps for S1, and 329 steps for S2. MAS were evaluated prior to donning the equipment and after doffing the equipment.

## Results

**Figure 6:**
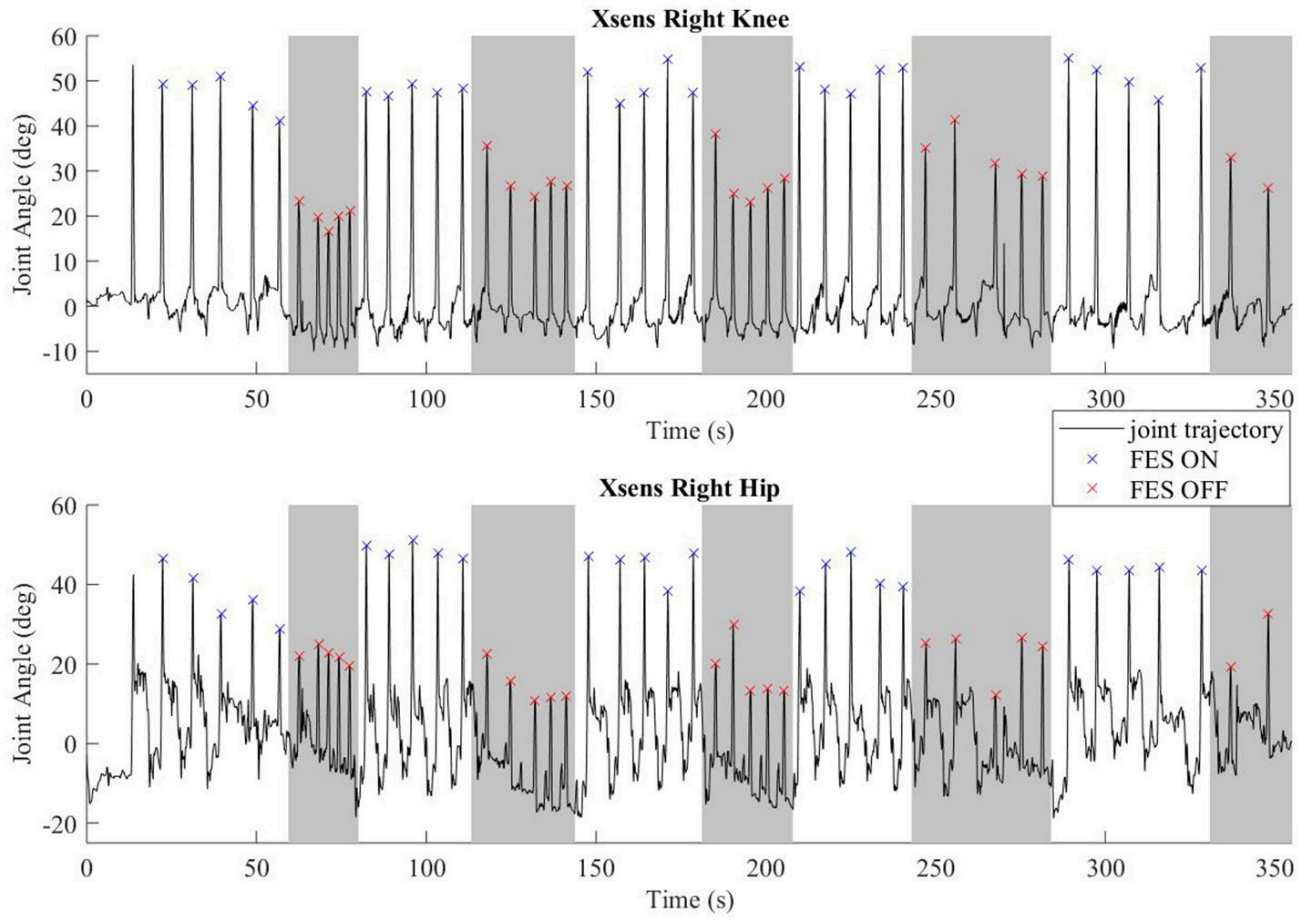
Representative joint angles from walking trial with subject S2. The gray bars indicate periods with FES off and the white bars indicate steps taken with FES on. Flexion peaks are identified by hash marks; blue for steps with FES assistance, red for steps without FES assistance.

Common peroneal stimulation increased the step length for both subjects, which was reflected in the kinematic data. The hip and knee angles of a representative walking session is plotted in Figure 6. The colored dots/hashes indicate the peaks found with a MATLAB algorithm, and does not include half steps, which are the steps in and out of neutral stance (when both hip equilibrium positions are equivalent). The gray bands indicate periods where FES is off. As seen in the data, the subject was able to achieve significantly greater hip and knee flexion with FES, which is indicative of larger steps and greater toe clearance.

**Figure 7:**
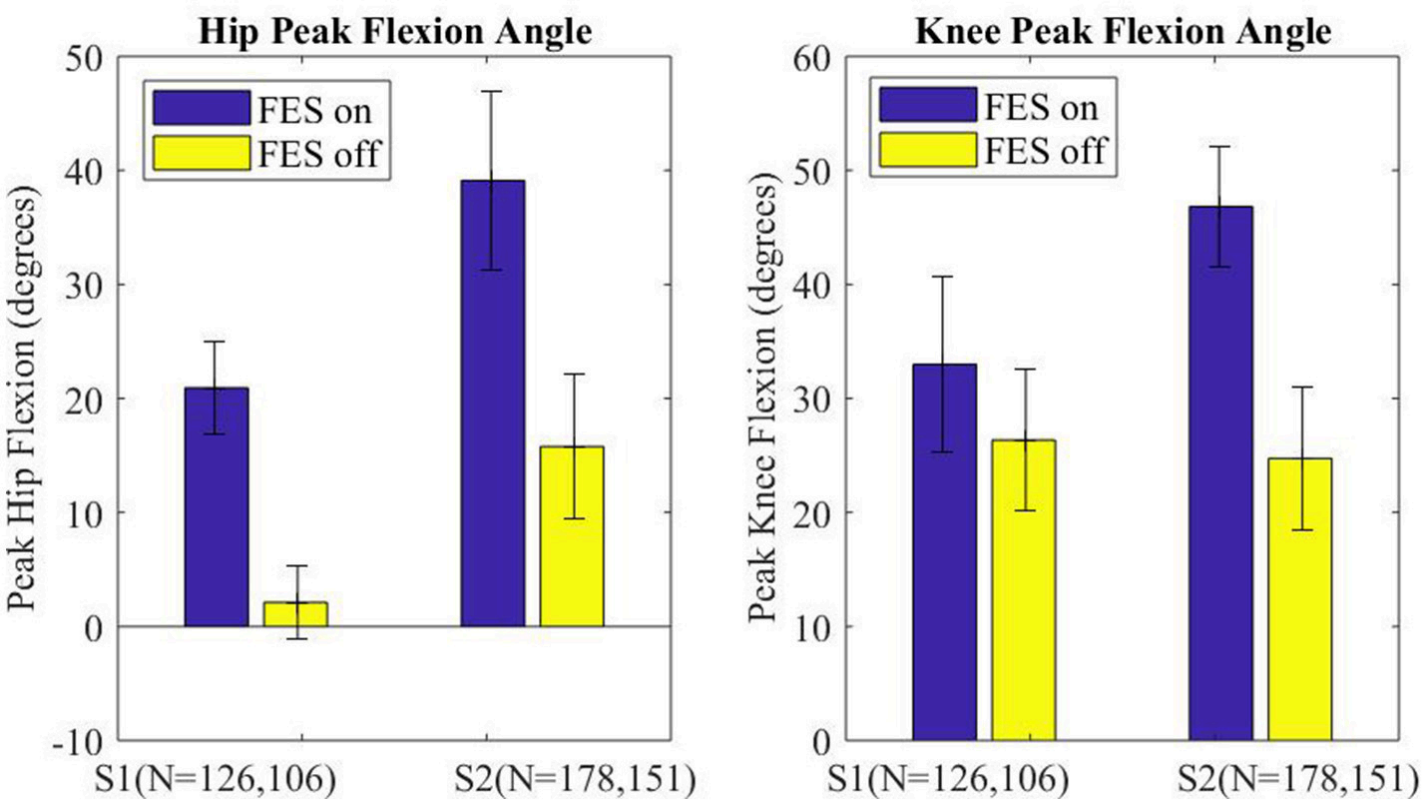
Median peak joint angle across all steps for each subject plotted for FES assistance on and FES assistance off. Error bars mark plus and minus half of the interquartile range. Wilcoxon analysis determines FES on and off had significantly different medians for each joint of each subject, *p* < 0.01.

The peak flexion angle during swing was parsed for each swing joint per step and grouped by FES on or off and by subject. The median peak flexion of all steps (right and left) for the hip and knee joints of each subject are plotted in Figure 7, with error bars denoting plus and minus half the interquartile range. Stimulation assistance increased the median hip peak flexion angles by 21.1° (236%) and the median peak knee angle by 14.4° (56%). Significance in differences in the peak flexion angles were assessed using the Wilcoxon tests for the intra-participant peaks, which indicated that the differences in peak knee and hip joint flexion between the FES-on and FES-off cases were significant with greater than a 99% confidence level. Wilcoxon was chosen due to the non-normality of the data as indicated by the Lilliefors test.

**Figure 8:**
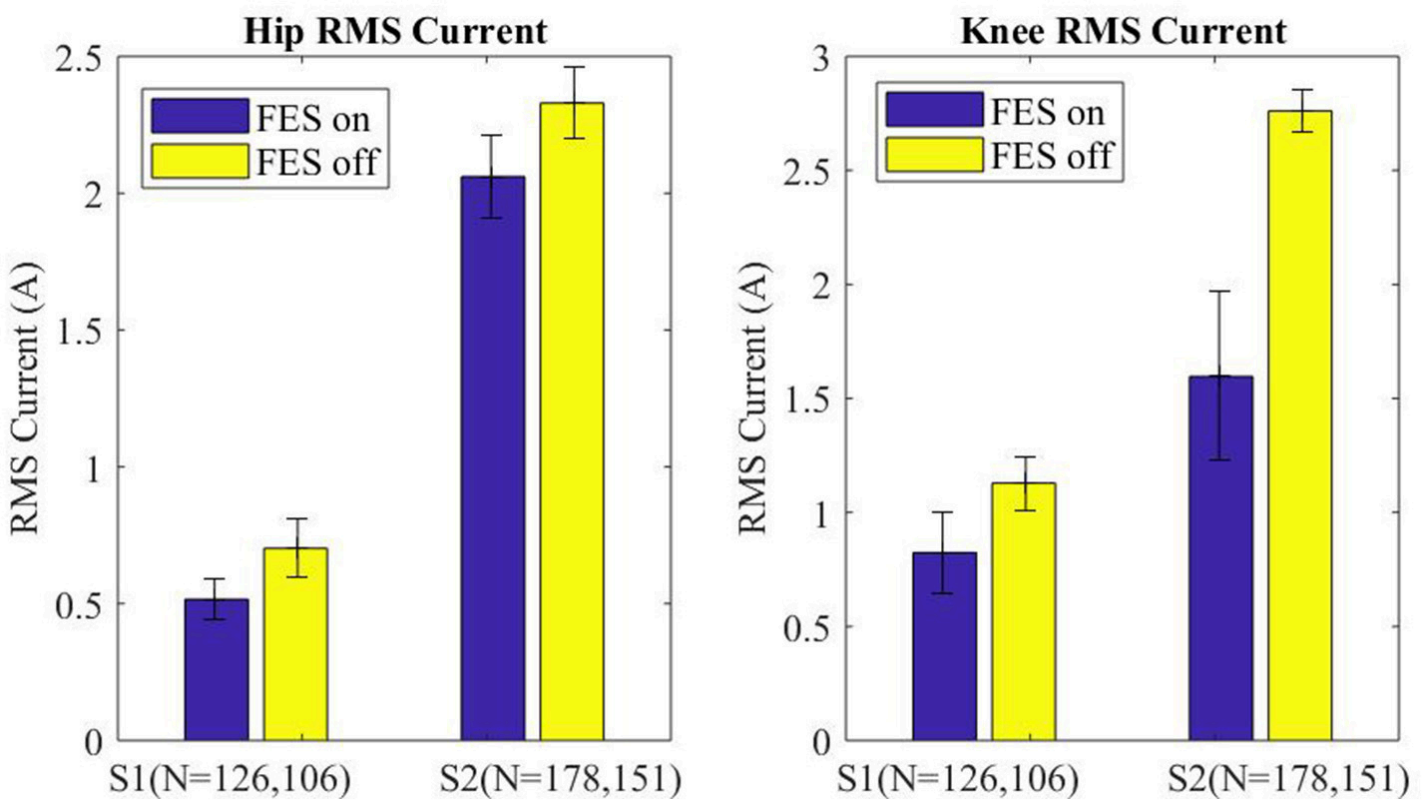
Median of the RMS current of the swing leg for all steps plotted for FES assistance on and FES assistance off. Error bars mark plus and minus half of the interquartile range. Wilcoxon analysis determines FES on and off are significantly different for each joint of each subject, *p* < 0.01.

Motor current data from the exoskeleton was logged and analyzed to evaluate the effects of FES assistance on motor torques. Specifically, RMS motor current provides a direct indication of how much effort is being exerted by the exoskeleton to move the paretic limbs. The RMS motor current for the swing leg was parsed for each step (right and left) and grouped into FES-on and FES-off for each subject. The median swing motor current plus and minus half of the interquartile range is shown in Figure 8 for each subject with FES on and off. Across subjects FES decreased the swing RMS current by 228 mA (15.0%) at the hip motors and 734 mA (37.8%) at the knee motors. Additionally, the intra-participant median swing RMS currents with FES-on and FES-off were significantly different with a 99% confidence level for both subjects, per Wilcoxon tests of the data.

## Discussion

Stimulation of the flexion withdrawal reflex improved the flexion kinematics significantly during exoskeleton assisted gait for participants with severe extensor spasticity. Qualitatively, both subjects had difficulty achieving toe clearance while walking with the exoskeleton without stimulation (i.e., toes consistently dragged on the ground). Adding stimulation greatly improved the gait kinematics and enabled these participants to take significantly larger strides.

Stimulation values were set while sitting and remained constant throughout the session that lasted over 1 h. Accommodation during the span of the trial was not assessed. It is possible that switching the FES off every ten steps effects accommodation. Continued research that specifically explores accommodation and varying FES amplitude should be performed. The stimulation amplitude was intentionally set below a level that would saturate the withdrawal reflex. Based on the experiments performed here, a relatively low-level of reflex stimulation appears to be sufficient to suppress extensor spasticity. A stronger reflex may have increased the observed effect, but may also increase habituation. FES duration during each step was established at a level that coincided with the exoskeleton flexion, accounting for the reflex delay ~100 ms; duration was not systematically evaluated. Continued studies that explore the physiological response (e.g., electromyography) of spasticity and peroneal stimulation is encouraged.

Additionally FES has been shown to provide therapeutic value for persons with SCI by activating the dormant neuromuscular tissue of the paralyzed limbs. The improved blood flow furthermore may reduce the likelihood of some comorbidities associated with SCI. For these reasons the addition of flexor withdrawal stimulation may be beneficial to all SCI individuals, including individuals with minimal spasticity.

Although some prior studies indicate a possible reduction in spasticity resulting from the use of exoskeletons, in this research, the MAS scores did not change significantly from before to after the walking session. The inherent limitations of the MAS test due to the coarse scoring and human dependent evaluation may attribute to unperceivable change in spasticity. Another consideration is severe spasticity may be more resistant to fatigue than mild spasticity. Furthermore, the focus of the study was acute spasticity (intra-step); a study that examines long term changes in spasticity should account for variables such as intervention duration, rest frequency and duration, and possibly include multiple MAS evaluators or other test for spasticity.

The significant reduction in the motor RMS current per step in conjunction with improved peak flexion is clear indication that extensor tone (spasticity) impedes the exoskeleton assisted flexion, and that flexor stimulation concerted with the exoskeleton flexion can restore a typical range of motion. The significance of the RMS current reductions is that the improved kinematics coincided with a reduction in exoskeleton effort, therefore, the improved flexion can be attributed exclusively to the FES of the common peroneal nerve. Electrical stimulation increased the electrical efficiency and improved performance by adding synergy between man and machine, muscles and motors.

Discrepancies between the exoskeleton kinematic data and the Xsens measured joint angles were perceivable suggesting that the human-robot interface (straps, soft tissue) allows for considerable deformation, especially when spasticity impedes exoskeleton motion. The Xsens provided successful means for motion capture of the user within the exoskeleton. Optical options for motion capture were not feasible due to the coverage of the exoskeleton, as well as the necessity of a very large motion capture arena for over-ground gait. Precaution was taken to securely fasten the Xsens sensors with elastic Velcro straps; trials where a Xsens sensor slipped substantially were not included.

A greater number of subjects should be evaluated to make generalized claims regarding the use of supplemental FES for subjects with severe extensor spasticity. Furthermore, the rehabilitation community would benefit from studies that explore the broader impact of exoskeletal walking in the SCI population.

## Conclusion

In two motor-complete-thoracic-level SCI subjects with severe lower limb extensor spasticity, supplemental stimulation of the common peroneal nerve during swing was shown to significantly enhance walking movements generated by the exoskeleton, and to significantly reduce the current demand on the exoskeleton motors. Results of more subjects would strengthen the conclusion of this work. The authors hope that employing supplemental FES as described here will make exoskeletal walking a viable option for individuals with SCI and severe extensor spasticity.

## Ethics statement

This study was carried out in accordance with the recommendations of the Vanderbilt Internal Review Board with written informed consent from all subjects. All subjects gave written informed consent in accordance with the Declaration of Helsinki. The protocol was approved by the Vanderbilt Internal Review Board.

## Author contributions

AE conceived and conducted the study, performed data analysis and wrote and edited the manuscript. MG coordinated the study and wrote and edited the manuscript.

### Conflict of interest statement

MG is an inventor on intellectual property that has been licensed by Vanderbilt University to the Parker Hannifin Corporation for use in the Indego exoskeleton. The other author declares that the research was conducted in the absence of any commercial or financial relationships that could be construed as a potential conflict of interest.
